# Impact of COVID-19 on anatomy education: a student-based survey and future perspectives

**DOI:** 10.3389/fmed.2026.1700678

**Published:** 2026-05-28

**Authors:** Kapil Kumar Malviya

**Affiliations:** Department of Anatomy, Institute of Medical Science, Banaras Hindu University, Varanasi, Uttar Pradesh, India

**Keywords:** anatomy education, COVID-19, medical education, online learning, survey

## Abstract

The COVID-19 pandemic affected every aspect of life worldwide, including the medical education system, with anatomy teaching being one of the most affected disciplines. To maintain continuity of education, educators rapidly transitioned to virtual teaching methods. The present study aimed to assess the effectiveness of virtual teaching methods during the pandemic by conducting a student-based survey (378 undergraduate and postgraduate medical students) and comparing them with traditional in-person teaching methods. Data were collected using a structured questionnaire designed to evaluate student’s preferences and perceptions regarding virtual teaching. The survey showed that PowerPoint presentations were the most preferred method for online theory teaching, with an average of ~89.00% (*n*=336) selecting this option across all streams. For online practical classes, YouTube dissection videos were chosen by ~50.00% (*n*=189) of students of all streams as the most suitable method for learning anatomical dissection online. In-person live lectures with live streaming were chosen as the best model of online anatomy teaching by most of the students, with an average of ~80.00% (*n*=302). Despite the adoption of various digital tools for online teaching, a significant proportion of students reported that their understanding through online teaching was lower compared to classical in-person teaching methods, with an average response of ~94.50%. Overall, the results indicate a general dissatisfaction among students with the fully online model of anatomy teaching; however, the responses also provided valuable suggestions for improvement of virtual teaching methods. The findings of this study highlight the challenges associated with online anatomy education and provide insights into potential strategies for improving virtual teaching methods. The data obtained may help educators to design more effective online teaching approaches, not only in situations such as pandemics but also as part of future blended learning models in medical education.

## Introduction

The COVID-19 pandemic caused unprecedented disruption to the global education system, including medical education, where anatomy teaching was particularly affected due to its reliance on hands-on training and in-person teaching modalities (1–4). Anatomy education is a fundamental pillar of medical education, and cadaveric dissection is a critical component for understanding human anatomy (5). Traditionally, anatomy teaching integrates multiple modalities, including synthetic models of different body parts, bones, digital tools such as anatomical software and atlases, and prosected specimens (6). During the pandemic, medical institutions rapidly transitioned to online teaching formats to ensure continuity of education. The shift included the adoption of virtual lectures, problem-based learning, pre-recorded lectures, modified mentoring systems, and online assessments such as online written exams, quizzes, assignments, and viva examinations conducted via video conferencing platforms (7–14). While these measures enabled uninterrupted teaching, they also resulted in significant limitations, particularly the absence of cadaveric dissection and access to various pathological specimens, radiological specimens, models, skeletons, and others (15–20). Simultaneously, reduced body donation during the pandemic further affected cadaveric dissection and anatomy model preparation, necessitating new advanced techniques in the anatomy education curriculum (21–28). The main facilitator of the online learning model was the use of the internet, along with the effective integration of online teaching platforms, new technologies, and pedagogical approaches. Several studies have evaluated the impact of online teaching across various medical disciplines, which can be categorized as follows:

1. *Impact of COVID-19 on anatomy teaching*: Herr and Nelson (18) surveyed dental students and described the effect of online teaching. Ramos-Morcillo et al. (29) and Puljak et al. (30) performed a survey among nursing students to explain the challenges faced by them during COVID-19 (29, 30).
2. *Student satisfaction with online tools*: A study on Ayurvedic students by Sawarkar et al. (31) described the introduction of online teaching, its challenges, and its effects. Obrero-Gaitán et al. (32) described an innovative approach to teaching neuroanatomy using 3D models for physiotherapy and nursing students, along with the challenges faced in teaching neuroanatomy and using such models during the COVID-19 pandemic.
3. Transition challenges: Rossettini et al. (33) highlighted the importance of the face-to-face education model for physiotherapy students and the difficulties they faced with online learning during COVID-19 (33). All the studies suggested the need for continuity of medical education during the pandemic through online platforms; however, students across disciplines, including nursing, dental, and Ayurveda, expressed a preference for in-person teaching methods for skill and knowledge development. Considering the pandemic and similar situations, students favored a blended learning model integrating both in-person and online methods.

In the initial stage of the pandemic, feedback from medical students and faculty members was collected to improve teaching quality, address shortcomings, and fill existing gaps. The American Association of Clinical Anatomists’ online member forum proposed strategies to improve anatomy education (34–37). However, there is limited literature available that provides insight into the difficulties faced during the pandemic, which could help explore future prospects and opportunities for improvements in anatomy theory and practical education (15, 18, 23, 38–42).

Despite these insights, there remains a limited comprehensive understanding of students’ experiences across diverse medical streams, particularly in relation to both theoretical and practical components of anatomy education during the pandemic. In this context, the present study aims to evaluate the impact of pandemic-induced changes in anatomy teaching on students from various medical streams, focusing on both theory and practical learning experiences. The study also seeks to analyse students’ perceptions of different online teaching methods and identify areas for improvement. By incorporating feedback from a diverse group of learners, the study aims to contribute to the development of more effective, flexible, and student-centered teaching strategies, particularly for use in emergency situations and future blended learning models.

## Methodology

### Study design and participants

The study was student-based and included 378 participants. A convenience sampling technique was used, targeting students enrolled in anatomy courses during the academic years 2019–2021, including first-year undergraduate and postgraduate students at the Institute of Medical Sciences, Banaras Hindu University, Varanasi, India. Participants included students from Bachelor of Medicine and Bachelor of Surgery (MBBS), Bachelor of Dental Surgery (BDS), nursing, Bachelor of Occupational Therapy and Bachelor of Physiotherapy (BOT-BPT), and post-graduate Ayurvedic programs.

#### Inclusion criteria

The inclusion criteria included students enrolled in MBBS, BDS, nursing, BOT-BPT, and postgraduate Ayurveda programs who attended online anatomy classes during the pandemic and provided informed consent.

#### Exclusion criteria

The exclusion criteria included students who did not study anatomy through online mode during the pandemic and those with incomplete responses. Since the study was exploratory in nature, a formal power analysis was not conducted. However, the sample size of 378 covered ~80–90% of the undergraduate and postgraduate anatomy students at the institution, providing considerable coverage.

### Questionnaire development and validation

The study assessed the effect of online teaching methods using a questionnaire developed based on prior literature and experiences of online anatomy teaching during the pandemic (34). For content validity, the questionnaire was assessed by two subject experts, and minor revisions suggested by them were included. A pilot study was performed with 10 students (not included in the main sample) to evaluate the comprehensibility of the questionnaire. Feedback from the pilot group was used to refine the questionnaire.

### Ethical approval and consent

The Institutional Ethical Review Committee approved the study at Banaras Hindu University, Varanasi, India. A separate consent form was provided to each participant along with the questionnaire, and, after obtaining consent from each participant, the responses to the questions were collected.

### Questionnaire structure

The questionnaire comprised 15 questions related to anatomy education during the COVID-19 pandemic. It included a combination of categorical, multiple-choice, multiple-response, and open-ended descriptive questions. Descriptive responses were systematically categorized and coded for analysis. Certain questions permitted the selection of more than one response, while others were designed to capture detailed narrative input. No Likert-type scales were used in the questionnaire.

The complete questionnaire is provided as Supplementary material.

### Survey distribution and data collection

The survey was distributed through face-to-face interaction with students. Each completed questionnaire was reviewed individually by the author, and responses were screened for completeness. Data analysis was performed as described below:

### Data analysis

Although the survey was administered in person, no personally identifiable information was used during data processing, and the results were analyzed and reported in aggregated form only. Participation was voluntary, and students were informed that their responses would not influence academic evaluation. The questionnaire (Questions 6–15) and corresponding responses were categorized into six themes to ensure structured analysis and to enhance clarity and comprehensibility of the data.

#### Quantitative data analysis

Participants were categorized into MBBS (*n* = 129), BDS (*n* = 73), nursing (*n* = 73), BOT-BPT (*n* = 66), and postgraduate Ayurvedic (*n* = 37) students. Survey responses for teaching modalities used in online theory lectures were divided into PowerPoint presentations (PPTs), YouTube videos, anatomy software, eBooks, and social media platforms. The preferred methods for delivering lectures were divided into in-person lectures with or without live streaming, previously recorded lectures, and other lecture formats. Similarly, survey responses for modalities used to deliver online practical classes were divided into YouTube dissection videos, 3D live dissection videos, 3D models, histology atlases, and dissection atlases.

#### Qualitative data analysis

Responses to the open-ended questions were coded, and descriptive codes were applied to summarize the content (43). Each narrative question was assigned a unique code for analysis. The codes were developed after analyzing the answers, considering the common answers and problems, and grouping similar answers into a single code. Responses to questions describing the drawbacks of online teaching during COVID-19 were categorized into eight different codes. The future prospects of the study were divided into two subcategories: Theory lecture improvement-related responses and practical improvement-related responses. The teaching methods used for both are different, so the two subcategories were coded separately. The theory lecture subcategory was assigned eight codes, while the practical subcategory was assigned 10 codes. For the open-ended questions, consistency was ensured through independent coding by two reviewers. A hybrid approach combining both deductive and inductive coding was used. Responses were initially categorized based on existing literature, and subsequently, new themes were added (34). Discrepancies were resolved through discussion until a consensus was reached.

### Statistical analysis

To address differences across student categories (MBBS, BDS, nursing, BOT-BPT, and postgraduate Ayurveda), a more robust inferential statistical analysis was performed. Specifically, the chi-squared goodness-of-fit test was used to assess significant differences in categorical responses. MS Excel was used for data compilation, and GraphPad Prism 8 was used to plot the data.

## Results

The present study was student-based and aimed to assess the effect of the COVID-19 pandemic on anatomy education. Traditional teaching methods had to be shifted to an online education model to maintain the continuity of the educational process during the pandemic. Various advanced modalities were implemented for online anatomy teaching in medical schools, including 3D models, animation videos, YouTube videos, cadaveric dissection videos, and anatomy practical videos. The study discusses the drawbacks and future prospects of virtual anatomy education, as well as the methods used to improve both online theory and practical teaching. It also compares virtual anatomy education with traditional anatomy teaching methods.

There were 378 total participants in this study, of whom 172 were female (45.50%) and 206 were male (54.50%). The study population included MBBS undergraduates (34.12%) (*n* = 129), BDS students (19.31%) (*n* = 73), nursing students (19.31%) (*n* = 73), BOT-BPT students (17.46%) (*n* = 66), and postgraduate Ayurvedic students (9.80%) (*n* = 37).

In questions 1, 2, and 3, students were asked about their name, age/sex, and institution, respectively. Names were collected to avoid duplication of responses and to ensure authenticity, as the survey was conducted in person. However, this may introduce bias, and future studies should use anonymous surveys. Face-to-face administration ensured a high response rate, allowed instant clarification of doubts, and also included students with limited internet access. Age and sex data were collected to describe participant demographics and to explore changes in perception across varied groups in future studies. Institutional details were recorded to confirm the study’s target population.

In question 4, students were asked whether they were taught anatomy during the COVID-19 pandemic. All participants responded “yes” to this question. Question 5 was about the mode of teaching during the COVID-19 pandemic, and all participants involved in the study responded “online” to it.

### Theme 1: online teaching modalities and digital tools used

**Figure 1:**
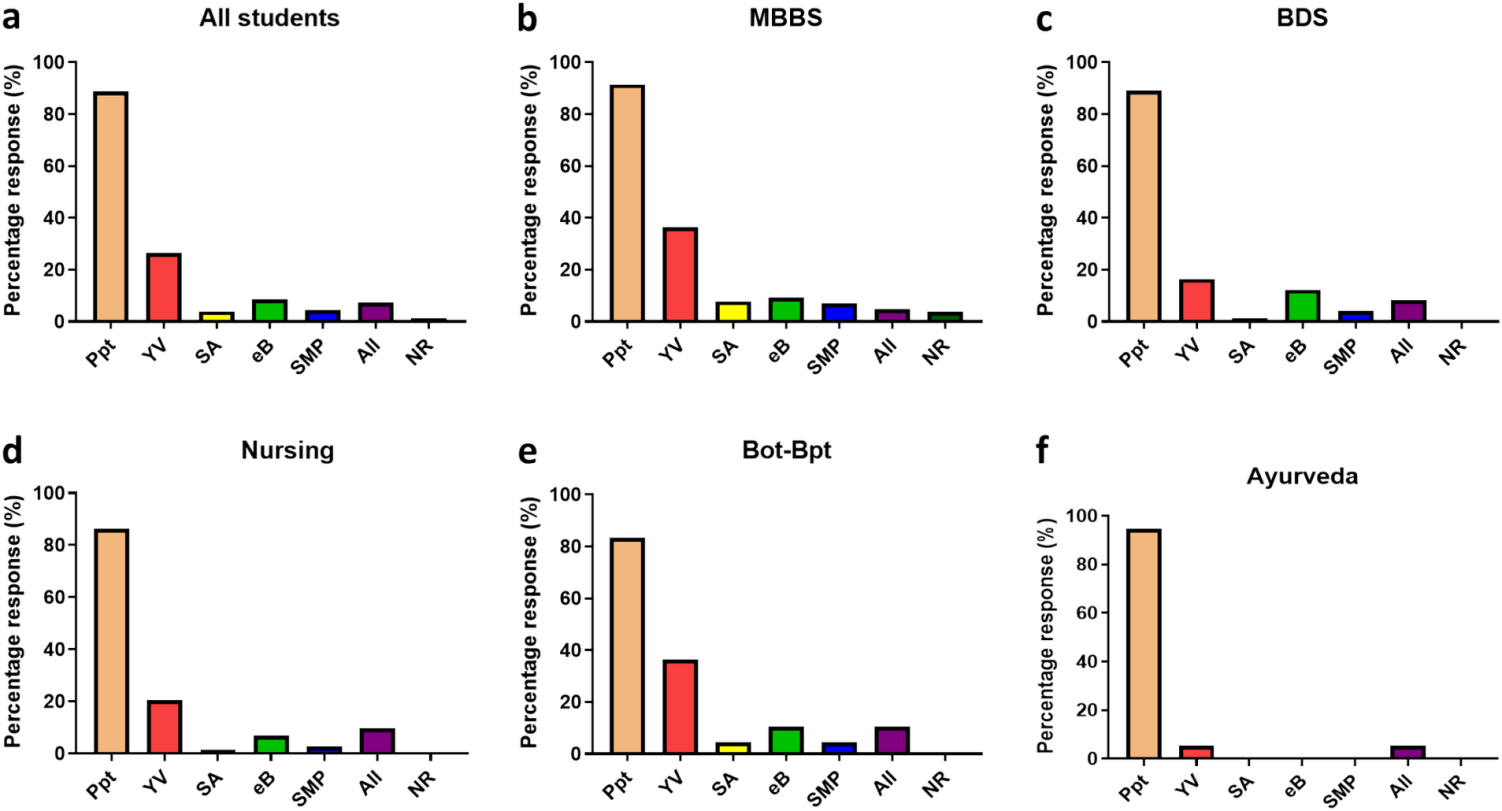
Students’ responses (in percentage) to approaches used for online anatomy theory teaching classes. Responses from all students **(a)** and individual courses—MBBS **(b)**, BDS **(c)**, nursing **(d)**, BOT-BPT **(e)**, and Ayurveda **(f)**—were recorded. The chi-squared goodness-of-fit test showed that the distribution differed significantly (*p* < 0.0001). MBBS, Bachelor of Medicine and Bachelor of Surgery; BDS, Bachelor of Dental Surgery; BOT, BPT, Bachelor of Occupational Therapy and Bachelor of Physiotherapy; PPT, PowerPoint presentation; YV, YouTube videos; SA, Software of Anatomy; B, eBooks; SMP, Social media platform; All, All of the above; NR, No response.

Evaluation of responses regarding online theory teaching modalities revealed that PowerPoint presentations were the most commonly used lecture method, selected by 88.89% of students, followed by YouTube videos (26.46%), reflecting the use of video-based learning resources. Other teaching modalities were used to a much lesser extent, including eBooks (8.70%), social media platforms (4.50%), and anatomy software (3.97%). All methods combined was selected by 7.41% of students, while 1.32% of students gave no response (Figure 1a). Importantly, the overall pattern across different student streams —MBBS, BDS, nursing, BOT-BPT, and Ayurveda—was similar, as depicted in Figures 1b-f. Statistical analysis using the chi-squared goodness-of-fit test showed that the distribution differed significantly (*p* < 0.0001).

**Figure 2:**
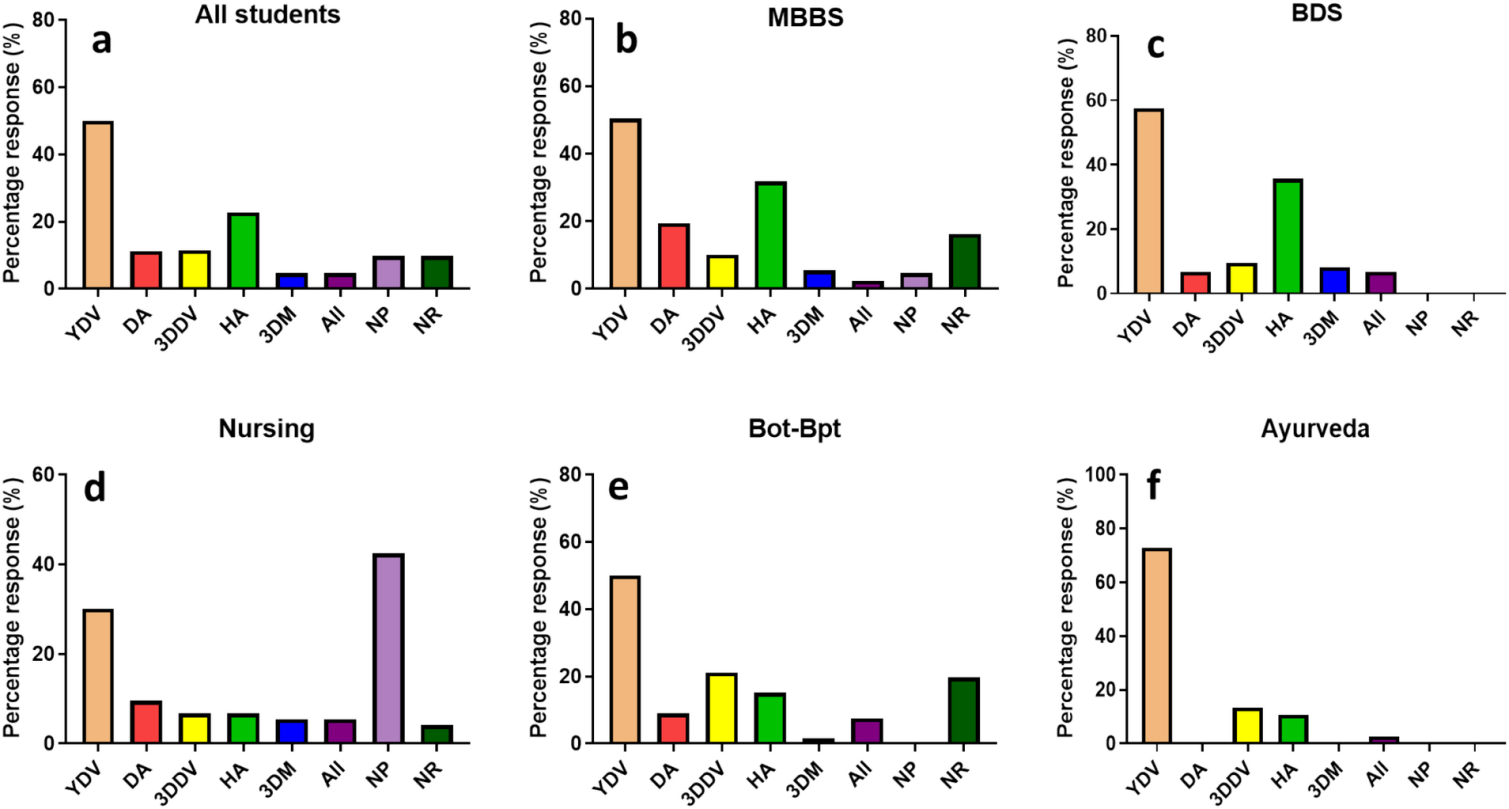
Students’ responses (in percentage) to approaches used for online anatomy practical teaching classes. Responses from all students **(a)** and individual courses—MBBS **(b)**, BDS **(c)**, nursing **(d)**, BOT-BPT **(e)**, and Ayurveda **(f)**—were recorded. The chi-squared goodness-of-fit test showed that the distribution differed significantly (*p* < 0.0001). MBBS, Bachelor of Medicine and Bachelor of Surgery; BDS, Bachelor of Dental Surgery; BOT-BPT, Bachelor of Occupational Therapy and Bachelor of Physiotherapy; YUV, YouTube dissection videos; DA, Dissection atlas; 3DDV, 3D dissection videos; HA, Histology atlas; 3DM, 3D models; All, All of the above; NP, No practical; NR, No response.

Regarding online practical teaching modalities, YouTube dissection videos were the most commonly used approach, reported by 50.00% of students. The histology atlas (22.75%) emerged as the second most commonly used approach, highlighting the importance of microscopic anatomy in online teaching. Other approaches included dissection atlases (11.36%), 3D dissection videos (11.64%), and 3D models (4.76%), respectively (Figure 2a). A subset of students (4.76%) selected all the given approaches, whereas 9.79% indicated that no online practical classes were conducted, and an additional 9.79% did not respond to the question (Figure 2a). Among students from different streams, YouTube dissection videos were the most commonly used modality, particularly among Ayurveda (73.00%) and BDS (58.00%) students. The histology atlas was the second choice for MBBS and BDS students. All other options showed lower percentages, as shown in Figures 2b–f. Statistical analysis using the chi-squared goodness-of-fit test showed that the distribution differed significantly (*p* < 0.0001).

### Theme 2: preferred online teaching models and class organization

**Figure 3:**
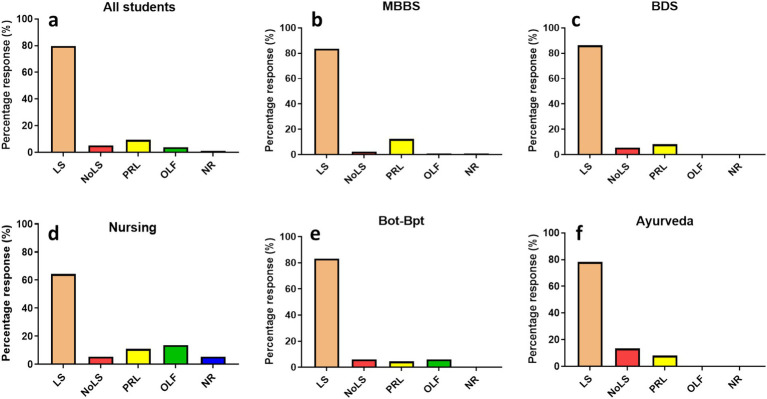
Students’ responses (in percentage) for the most suitable model for anatomy teaching through online lectures. Responses from all students **(a)** and individual courses—MBBS **(b)**, BDS **(c)**, nursing **(d)**, BOT-BPT **(e)**, and Ayurveda **(f)**—were recorded. The chi-squared goodness-of-fit test showed that the distribution differed significantly (*p* < 0.0001). MBBS, Bachelor of Medicine and Bachelor of Surgery; BDS, Bachelor of Dental Surgery; BOT-BPT, Bachelor of Occupational Therapy and Bachelor of Physiotherapy; LS, In-person live lectures with live streaming; NoLS, In-person live lectures without live streaming; PRL, Previously recorded lectures; OLF, Other lecture format; NR, No response.

Students were asked to choose the most suitable model for online anatomy teaching during the COVID-19 pandemic. Among all participants, in-person live lectures with live streaming were the most commonly preferred option (79.89%), indicating a strong inclination of students toward real-time interaction despite the virtual format. This was followed by previously recorded lectures (9.52%), in-person live lectures without live streaming (5.29%), and other lecture formats (3.97%), and 1.32% of students did not respond (Figure 3a). This trend remained consistent across all disciplines, where MBBS (83.72%), BDS (86.30%), BOT-BPT (83.33%), nursing (64.38%), and Ayurveda (78.38%) students favored in-person live-streamed sessions. Previously recorded lectures were the second choice across most disciplines, followed by other options, as shown in Figures 3b–f. Statistical analysis using the chi-squared goodness-of-fit test showed that the distribution differed significantly (*p* < 0.0001).

**Figure 4:**
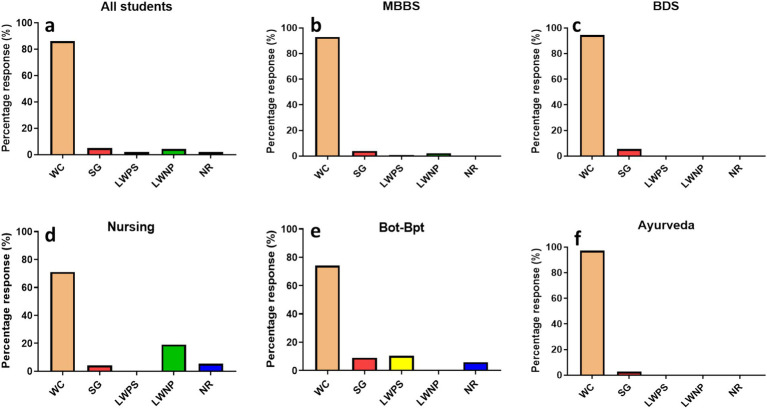
Students’ responses to online lectures and practical classes conducted in small groups or as a whole class. Responses from all students **(a)** and individual courses—MBBS **(b)**, BDS **(c)**, nursing **(d)**, BOT-BPT **(e)**, and Ayurveda **(f)**—were recorded. The chi-squared goodness-of-fit test showed that the distribution differed significantly (*p* < 0.0001). MBBS, Bachelor of Medicine and Bachelor of Surgery; BDS, Bachelor of Dental Surgery; BOT-BPT, Bachelor of Occupational Therapy and Bachelor of Physiotherapy; WC, Whole class; SG, Small group; LWPS, Lectures as a whole group and practicals in small groups; LWNP, Lectures as a whole group and no practical; NR, No response.

Regarding the group organization of online anatomy classes, the majority of students (86.24%) reported that theory and practical anatomy classes were conducted as a whole group during the pandemic. Other responses included small-group teaching (5.03%), lectures conducted as a whole group with no practical sessions (4.50%), and lectures conducted as a whole group with practical sessions in small groups (2.12%) (Figure 4a). A small proportion of students (2.12%) did not respond to the question (Figure 4a). Whole-group teaching for both theory and practical sessions emerged as the dominant method across most disciplines, particularly among Ayurveda (97.30%), BDS (94.50%), and MBBS (93.00%) students. In contrast, small-group teaching was chosen by a smaller percentage of students (≤10%), mainly BOT-BPT students. Detailed percentages of other modalities for theory and practical classes across various streams are provided in Figures 4b–f. Statistical analysis using the chi-squared goodness-of-fit test showed that the distribution differed significantly (*p* < 0.0001).

### Theme 3: perceived effectiveness of online anatomy teaching

**Figure 5:**
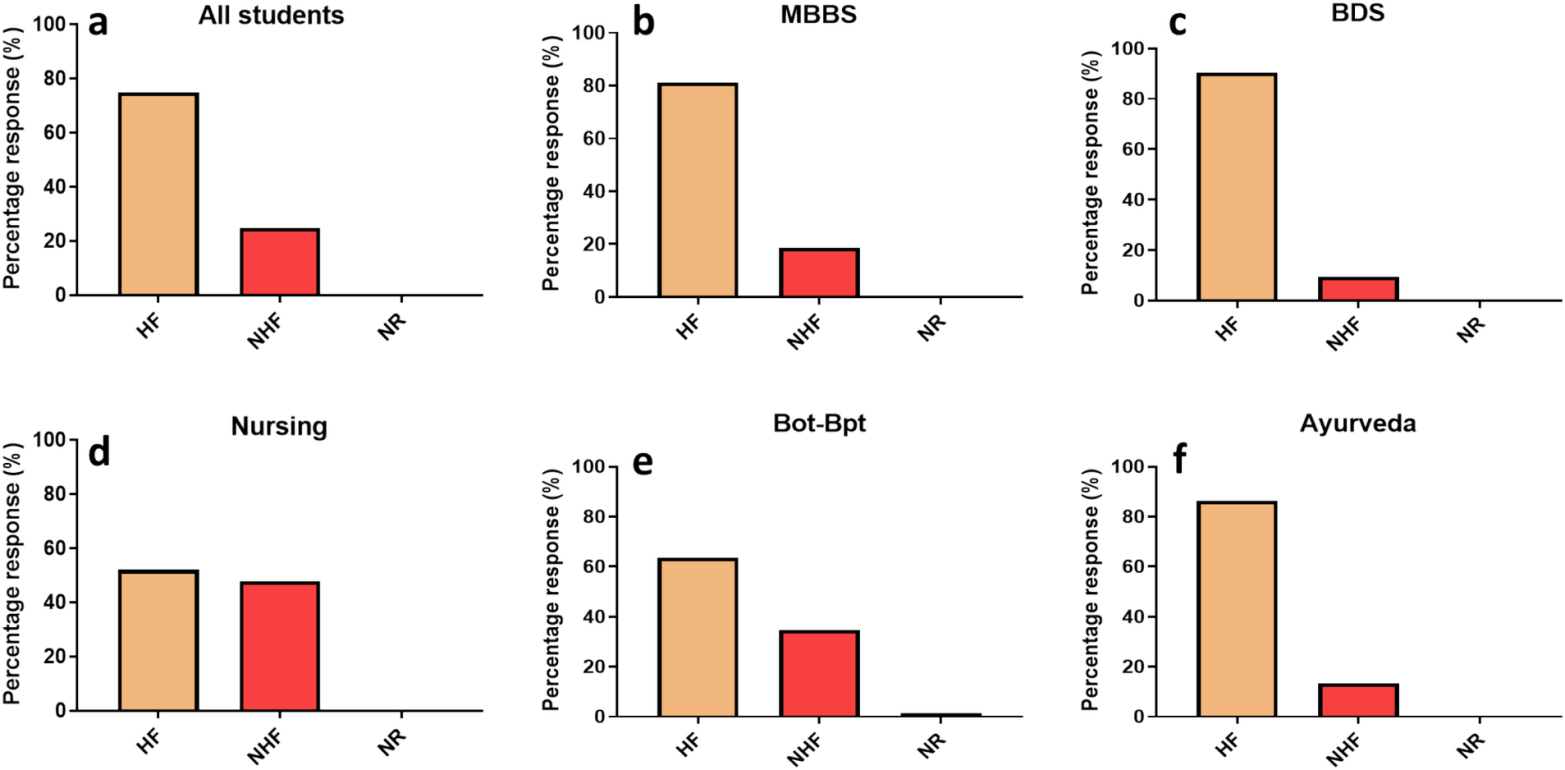
Students’ responses to the methods mentioned in questions 6, 7, and 8 regarding their helpfulness in teaching during COVID-19. Responses from all students **(a)** and individual courses—MBBS **(b)**, BDS **(c)**, nursing **(d)**, BOT-BPT **(e)**, and Ayurveda **(f)**—were recorded. The chi-squared goodness-of-fit test showed that the distribution differed significantly (*p* < 0.0001). MBBS, Bachelor of Medicine and Bachelor of Surgery; BDS, Bachelor of Dental Surgery; BOT-BPT, Bachelor of Occupational Therapy and Bachelor of Physiotherapy; HF, Helpful; NHF, Not helpful; NR, No response.

Students’ responses regarding the helpfulness of online lectures and practical classes showed that the majority of students (74.87%) perceived the online approaches and teaching models adopted for anatomy theory and practical classes during the pandemic as helpful (Figure 5a). Across different disciplines, BDS (90.40%) and Ayurveda (86.50%) students reported the highest levels of satisfaction with online teaching modalities, followed by MBBS students (81.40%). In contrast, nursing students showed the lowest level of satisfaction with teaching modalities (52.00%). Detailed percentages across all streams are provided in Figures 5b–f. The chi-squared goodness-of-fit test showed that the distribution of student responses (Helpful (HF), not helpful (NHF), and no response (NR)) differed significantly from equal expected proportions (*p* < 0.0001 for all groups). This reflects a strong preference for HF responses across cohorts, with minimal or absent NR.

**Figure 6:**
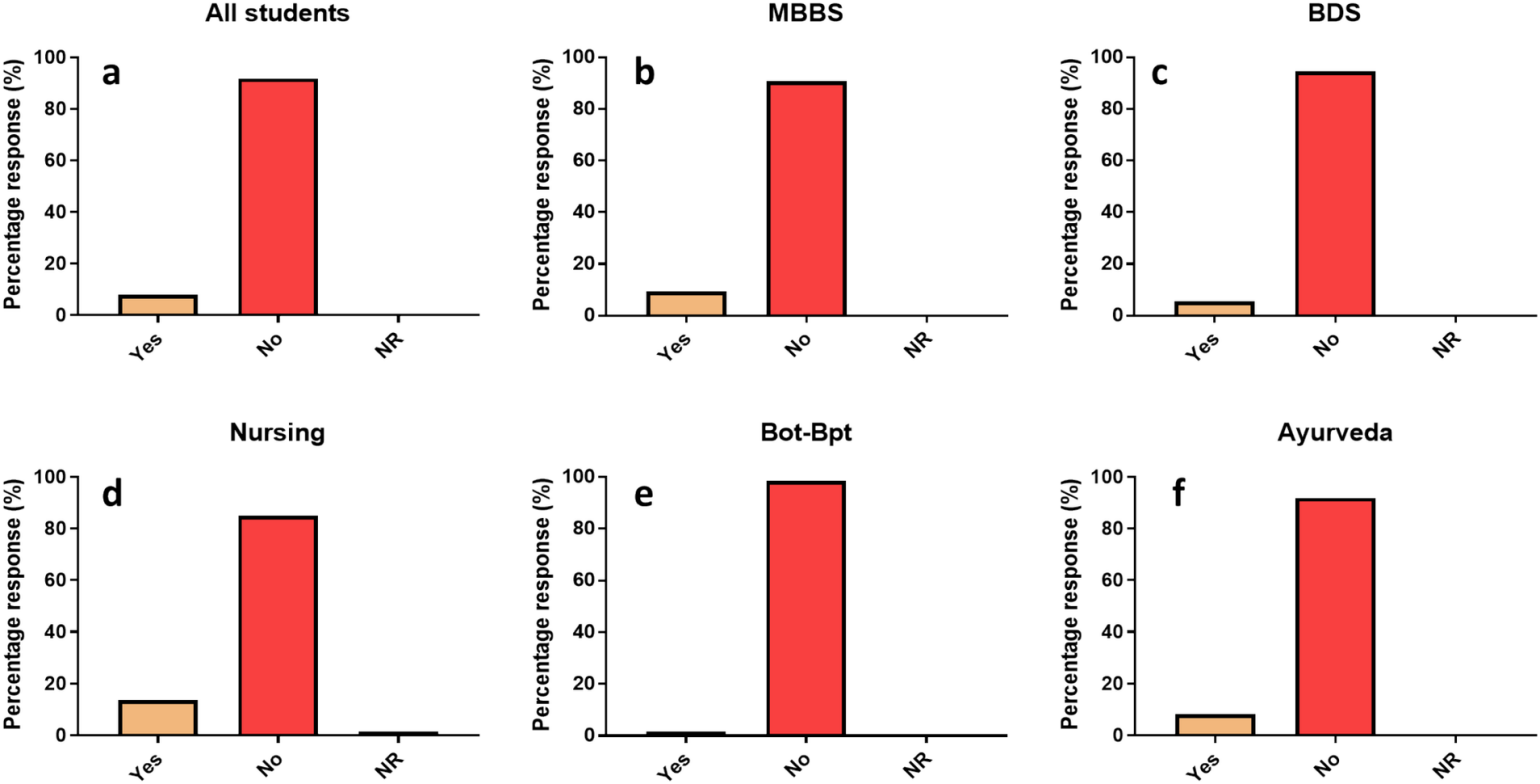
Students’ responses comparing online platforms for theory classes with traditional in-person teaching classes. Responses from all students **(a)** and individual courses—MBBS **(b)**, BDS **(c)**, nursing **(d)**, BOT-BPT **(e)**, and Ayurveda **(f)**—were recorded. The chi-squared goodness-of-fit test showed that the distribution differed significantly (*p* < 0.0001). MBBS, Bachelor of Medicine and Bachelor of Surgery; BDS, Bachelor of Dental Surgery; BOT-BPT, Bachelor of Occupational Therapy and Bachelor of Physiotherapy; NR, No response.

When comparing online theory education with pre-pandemic face-to-face anatomy lectures, the majority of students (91.80%) across all disciplines favored the traditional face-to-face method as being more effective and easier to understand than online teaching during the pandemic. Only 7.94% of students considered the online theory platform to be better than the previous traditional methods, indicating a strong preference for in-person lectures. The responses collected from individual groups, such as MBBS, BDS, nursing, BOT-BPT, and Ayurveda, are provided in Figures 6a–f.

**Figure 7:**
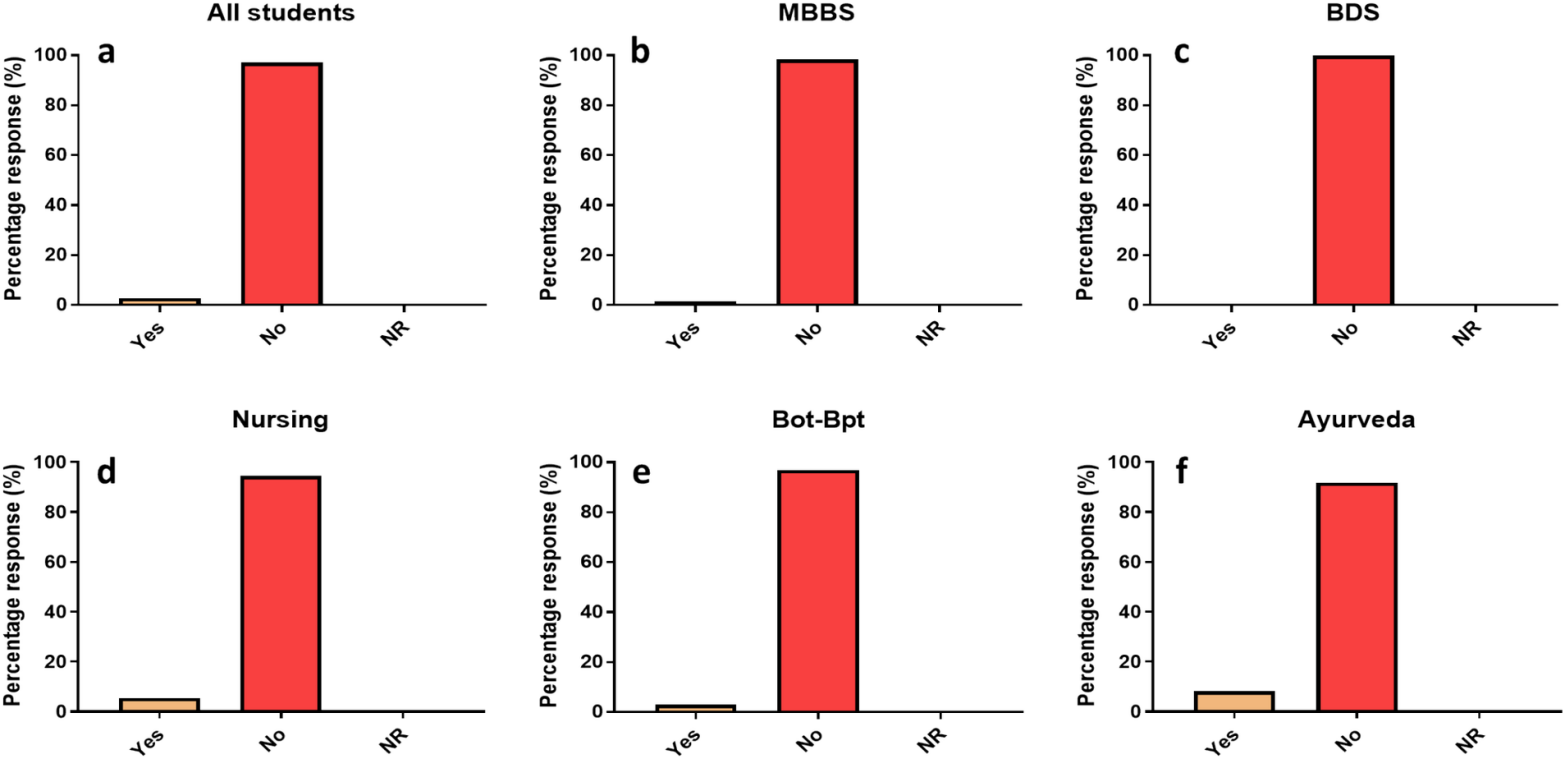
Students’ responses comparing online platforms for practical classes with traditional in-person practical classes. Responses from all students **(a)** and individual courses—MBBS **(b)**, BDS **(c)**, nursing **(d)**, BOT-BPT **(e)**, and Ayurveda **(f)**—were recorded. The chi-squared goodness-of-fit test showed that the distribution differed significantly (*p* < 0.0001). MBBS, Bachelor of Medicine and Bachelor of Surgery; BDS, Bachelor of Dental Surgery; BOT-BPT, Bachelor of Occupational Therapy and Bachelor of Physiotherapy; NR, No response.

Students from all disciplines reported that traditional offline practical methods provided better understanding and skill development compared to online methods. MBBS (98.45%) and BDS (100%) students showed a complete preference for traditional methods, highlighting the importance of real-time exposure. Detailed percentages for all student groups are provided in Figures 7a–f.

### Theme 4: challenges and limitations of online anatomy education

**Figure 8:**
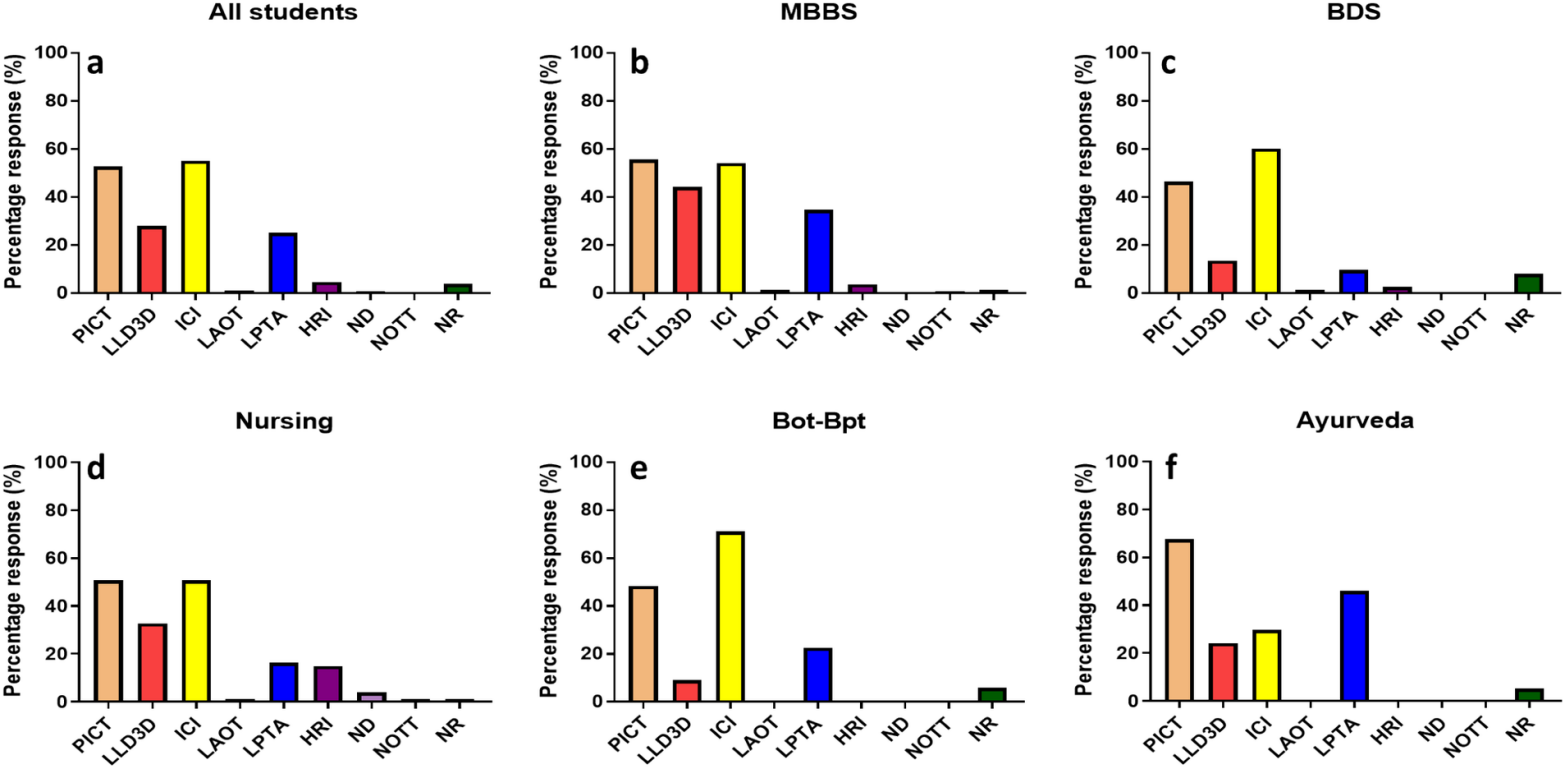
Students’ responses regarding the drawbacks of online teaching methods. Responses from all students **(a)** and individual courses—MBBS **(b)**, BDS **(c)**, nursing **(d)**, BOT-BPT **(e)**, and Ayurveda **(f)**—were recorded. The chi-squared goodness-of-fit test showed that the distribution differed significantly (*p* < 0.0001). MBBS, Bachelor of Medicine and Bachelor of Surgery; BDS, Bachelor of Dental Surgery; BOT-BPT, Bachelor of Occupational Therapy and Bachelor of Physiotherapy; PICT, Poor interaction and communication with teachers; LLD3D, Lack of live dissection and 3D models; ICI, Internet and connectivity issue; LAOT, Lack of awareness about online platforms; LPTA, Lack of a proper teaching ambience; HRI, Health-related issues; ND, No drawback; NOTT, No online time-to-time test; NR, No response.

Participants were asked about the drawbacks and issues of online teaching methods during the pandemic. Overall, the most frequently reported limitation was internet and connectivity issue (ICI), identified as the major problem related to online teaching methods by 55.29% of students, indicating infrastructural constraints. This was followed by less interaction and communication with teachers (52.91%), which hindered conceptual understanding (Figure 8a), clarification of doubts, and continuity of learning. Other prominent challenges included the lack of live dissection videos and 3D models (28.04%); the lack of a proper teaching ambience, resulting in poor motivation, reduced interest, and lower concentration (25.40%); health-related issues such as eyesight problems and headaches (4.76%), the lack of awareness about the online platform (1.06%), and the absence of online time-to-time tests (0.53%). According to 0.79% of students, online teaching had no drawbacks, while 3.97% did not respond to the question (Figure 8a). The chi-squared goodness-of-fit test revealed that the distribution differed significantly across all student streams (*p* < 0.0001 for every group). This indicates that students strongly emphasized certain drawbacks—such as poor interaction and communication with teachers (PICT), ICI, and lack of a proper teaching ambience (LPTA)—over other categories. Detailed responses from students across various streams are provided in Figures 8b–f.

### Theme 5: overall impact of COVID-19 on medical education

**Figure 9:**
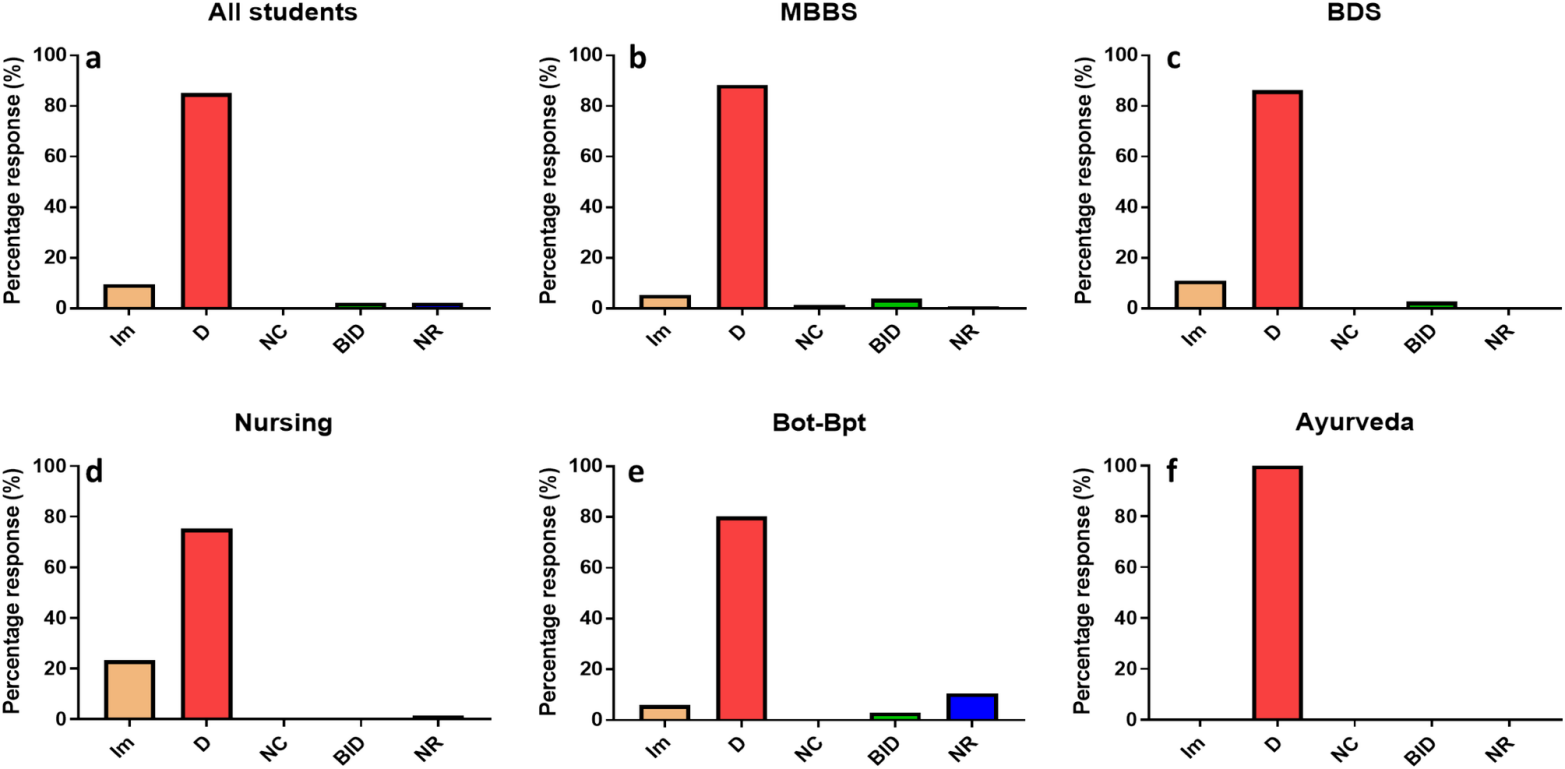
Students’ responses regarding the improvement or deterioration of medical education during COVID-19. Responses from all students **(a)** and individual courses—MBBS **(b)**, BDS **(c)**, nursing **(d)**, BOT-BPT **(e)**, and Ayurveda **(f)**—were recorded. The chi-squared goodness-of-fit test showed that the distribution differed significantly (*p* < 0.0001). MBBS, Bachelor of Medicine and Bachelor of Surgery; BDS, Bachelor of Dental Surgery; BOT-BPT, Bachelor of Occupational Therapy and Bachelor of Physiotherapy; IM, Improved; D, Deteriorated; NC, No change; BID, Both improved and deteriorated; NR, No response.

When students were asked whether medical education improved or deteriorated during the pandemic, most students (85.19%) reported a deterioration in the quality of medical education. In contrast, only 9.50% of students perceived an improvement in education. A smaller percentage answered that education had both improved and deteriorated (2.40%), while 0.50% observed no change and 2.40% did not respond (Figure 9a). Ayurveda students unanimously agreed (100%) that the quality of education had declined (Figure 9f), while nursing students demonstrated comparatively greater acceptance of the quality of education during the pandemic. The chi-squared goodness-of-fit test revealed that the distribution differed significantly across all student streams (*p* < 0.0001 for every group). Detailed responses from students of all streams are presented in Figures 9b–f.

### Theme 6: future directions and recommendations for improving online theory and practical teaching

**Figure 10:**
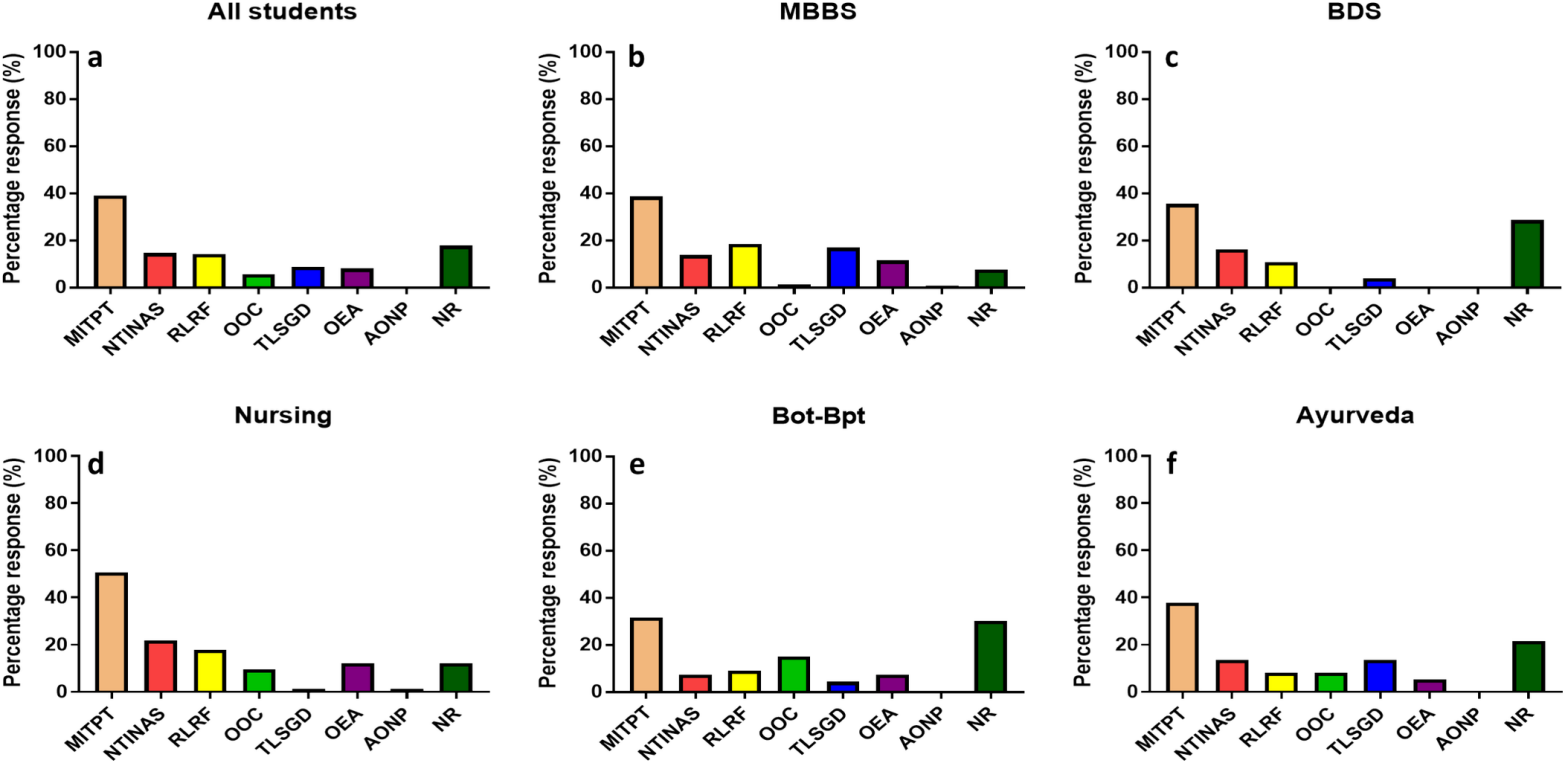
Students’ responses regarding improvements in online theory teaching for future implementation. Responses from all students **(a)** and individual courses—MBBS **(b)**, BDS **(c)**, nursing **(d)**, BOT-BPT **(e)**, and Ayurveda **(f)**—were recorded. The chi-squared goodness-of-fit test showed that the distribution differed significantly (*p* < 0.0001). MBBS, Bachelor of Medicine and Bachelor of Surgery; BDS, Bachelor of Dental Surgery; BOT-BPT, Bachelor of Occupational Therapy and Bachelor of Physiotherapy; MITPT, More interaction with teachers with a proper timetable of classes; NTINAS, Network and technical issues need to be resolved; new applications and anatomy software are required; RLRF, Recorded lectures for revision in the future; OOC, Only in-person classes; TLSGD, Theory lectures in small group discussions; OEA, Online exams or assignments; AONP, Awareness of online networks and platforms; NR, No response.

Various theory-based suggestions were provided by students when asked about the improvisation and improvement of online teaching in pandemic-like situations. The most frequently reported recommendation was increased interaction with teachers, supported by a well-structured and regular timetable, as cited by 39.15% of students (Figure 10a). The second most common suggestion was the improvement of network and technical issues, along with updated knowledge of advanced applications and anatomy software (14.81%). Access to recorded lectures for future revision was the third most common suggestion (14.28%) (Figure 10a). A smaller group of students suggested small-group theory discussions (8.99%), regular online assessments or assignments (8.20%), and a return to exclusively offline classes (5.82%) to improve online education. Better awareness and training regarding online platforms were suggested by a smaller number of students (0.53%), while 17.99% of students did not respond to this question (Figure 10a). Students from all streams also showed a similar trend for all suggestions, with slight variation in percentages. Detailed percentages across all streams are provided in Figures 10b–f. The chi-squared goodness-of-fit test showed that the distribution of students’ suggested improvements for future online anatomy theory teaching differed significantly from equal expected proportions across all academic streams (*p* < 0.0001).

**Figure 11:**
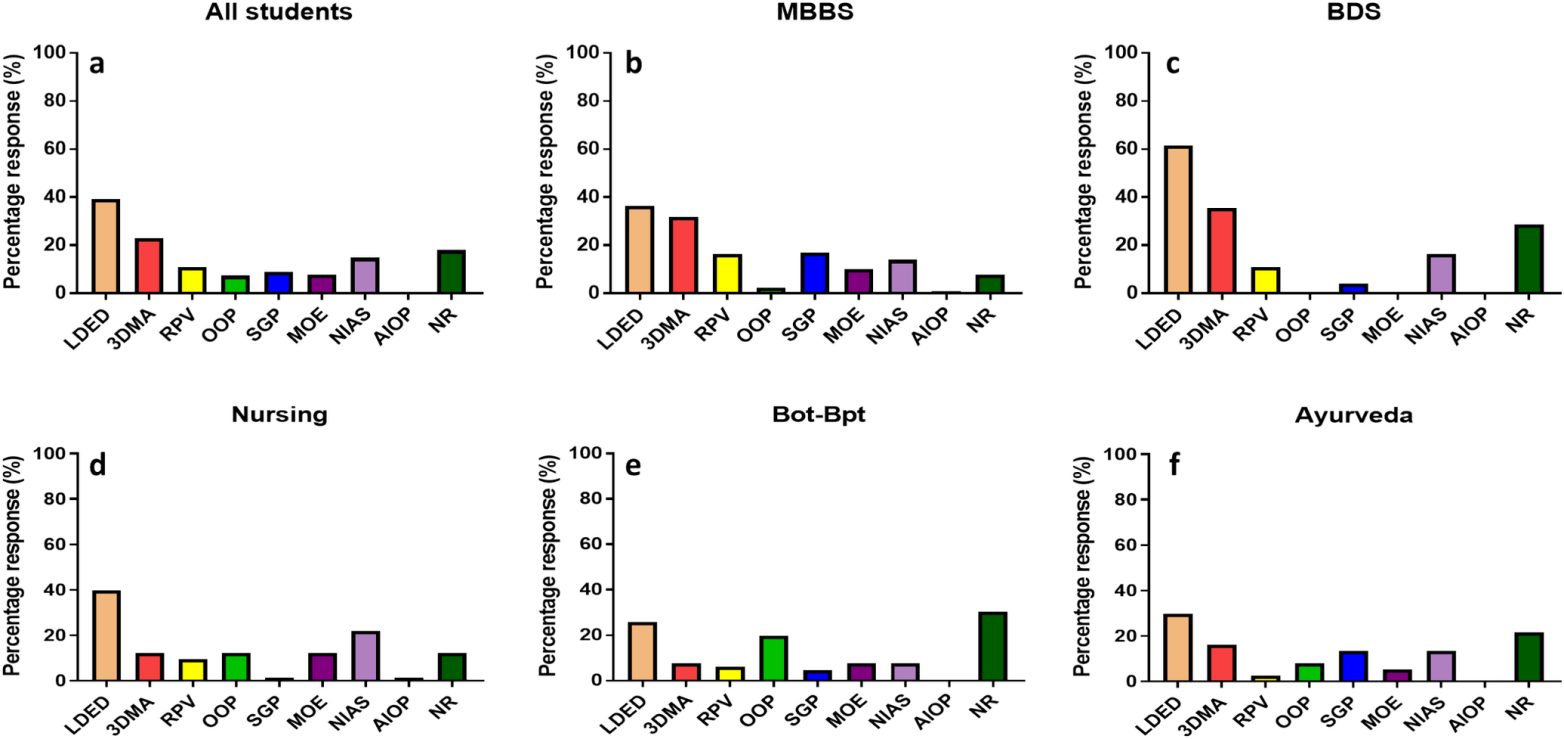
Students’ responses regarding improvements in online practical teaching for future implementation. Responses from all students **(a)** and individual courses—MBBS **(b)**, BDS **(c)**, nursing **(d)**, BOT-BPT **(e)**, and Ayurveda **(f)**—were recorded. The chi-squared goodness-of-fit test showed that the distribution differed significantly (*p* < 0.0001). MBBS, Bachelor of Medicine and Bachelor of Surgery; BDS, Bachelor of Dental Surgery; BOT-BPT, Bachelor of Occupational Therapy and Bachelor of Physiotherapy; LDED, Live dissection and more exposure to dissection; 3DMA, 3D models and animation; RPV, Recorded practical videos; OOP, Only in-person practicals; SGP, Small-group practicals; MOE, More online exams; NIAS, Network issues need to be resolved, and new anatomy software should be used; AIOP, Awareness about the internet and online platforms; NR, No response.

The most common suggestion was online live dissection and increased exposure to the dissection hall, selected by 39.41% of students (Figure 11a). The use of 3D models and animation was the second most preferred option, selected by 23.02% of students, followed by 14.81% of students who suggested resolving network issues and using new anatomy software (Figure 11a). Other responses included provision of recorded practical videos (10.85%), small-group practical sessions (8.99%), increased frequency of regular online practical examinations (7.67%), and a shift to offline-only practical classes (7.41%) (Figure 11a). A small proportion of students (0.53%) suggested the need for better awareness of online platforms, while 17.99% of students did not respond (Figure 11a). Students from different streams also showed a similar trend, prioritizing live dissection over other options. Detailed percentages across all streams are provided in Figures 11b–f. The chi-squared goodness-of-fit test showed that the distribution of students’ suggested improvements for future online anatomy practical classes differed significantly from equal expected proportions across all academic streams (*p* < 0.0001).

## Discussion

The study evaluated the effectiveness of online anatomy teaching during the COVID-19 pandemic across multiple medical disciplines. Discipline-wise categorization (MBBS, BDS, nursing, BOT-BPT, and Ayurveda) was adopted to account for differences in curriculum structure, teaching delivery, and the extent of practical exposure, all of which directly influence learning preferences and outcomes. This approach provides more meaningful insights into discipline-specific challenges; however, future studies across academic levels will be explored.

Online learning platforms have become increasingly popular in tertiary care medical institutions, enabling enhanced learning through visual, auditory, and verbal modalities (44–46). During the pandemic, online anatomy education used live online lectures, PPTs, YouTube videos, anatomy software, eBooks, and social media platforms (47). A key finding of the study was the predominance of PPTs, followed by YouTube videos, eBooks, social media platforms, and anatomy software, respectively. This preference can be attributed to the structured and controlled delivery of content in PPT, which closely mimics traditional classroom teaching and allows better cognitive organization of information (48). In contrast, YouTube, although the second choice and useful for providing creative visual content, lacks interactivity and limits active learning. eBook, as the third choice, offers advantages such as ease of accessibility, cost-effectiveness, and portability. Shigli et al. (49) described the role of PPT in undergraduate teaching as an effective tool for teaching and learning methods. The findings align with existing literature emphasizing that effective learning in medical education depends not only on content availability but also on guided instruction and interaction (23, 50, 51). The responses collected indicate that the popularity of PPT reflects the importance of structured pedagogy over purely resource-based learning.

During the COVID-19 pandemic, the disruption of anatomy practical teaching emerged as a critical concern, particularly regarding cadaveric dissection, histology practicals, and embryology (52–54). The reliance on plastic models, dissection atlases, YouTube dissection videos, 3D dissection videos, histology atlases, 3D models, and other digital substitutes highlights how institutions attempted to compensate for the absence of cadaveric dissection (20, 34, 55–60). For online practical teaching, YouTube dissection videos were the most popular choice due to their close resemblance to real-time cadaveric dissections, followed by histology atlases (61). The preference of Ayurveda and BOT-BPT students for 3D dissection videos further suggests that the depth of visualization becomes more important when hands-on exposure is lacking, particularly in disciplines with applied clinical relevance. Anatomy practicals were eliminated or shortened in nursing curricula to streamline medical education. However, this shift raises an important issue: While digital tools can supplement anatomy education, they cannot fully replicate the tactile, spatial, and experiential learning provided by cadaveric dissection (62).

Various teaching models were used to improve delivery outcomes for online teaching during the pandemic. In-person live lectures with live streaming were the most popular choice due to direct interaction between tutors and students. Students favored this model because it combines accessibility with immediate clarification of doubts, which is often missing in other formats. Previously recorded lectures were the second choice due to their flexibility and low internet dependency. MBBS and BDS students also preferred previously recorded lectures for repeated revisions due to the extensive anatomy syllabus. Ayurveda and BOT-BPT students selected in-person live lectures without live streaming as the second most popular choice. The results are in line with various studies, including Attardi et al. (62), which described the decrease in in-person lecture delivery during the COVID-19 pandemic. According to Cardall et al. (63), preclinical students prefer live online lectures when available, as online lectures allow greater acquisition of knowledge and information. The application of multimedia design can improve learning outcomes, and many research publications emphasize its integration into online medical education (64). According to previous literature, teaching through a combination of pre-recorded lectures and live-streaming methods helps students remain more focused and engaged (30, 65). This finding suggests an important pedagogical implication that integrated blended learning models, combining both synchronous (real-time interactive) and asynchronous (self-paced) instructional approaches, demonstrate greater effectiveness in enhancing learning outcomes compared to exclusively online teaching modalities.

According to the responses, whole-class teaching for both theory and practical sessions was the most preferred approach during the pandemic, while only a small percentage of students opted for small-group teaching. Interestingly, the preference for whole-class teaching over small-group teaching reflects practical constraints rather than pedagogical superiority. While small-group learning is known to enhance interaction and engagement, its implementation in an online setting is challenging due to logistical limitations and the need for structured scheduling. Recently, various research articles have described the effectiveness of large- and small-group online teaching. Srinivasan (66) described anatomy teaching through e-learning using large- and small-group discussions. Various types of audience response software, such as PollEv, Slido, Glisser, Crowdpurr, OMBEA, Pigeonhole Live, and TurningPoint, facilitate small-group teaching by enhancing student–teacher interaction (67). The findings suggest that technological and institutional readiness play a crucial role in determining the success of innovative teaching methods.

During the pandemic, virtual teaching was the only feasible method to continue medical education (68). The majority of students, including MBBS, BDS, BOT-BPT, and Ayurveda students, considered online anatomy education helpful for gaining knowledge during the pandemic. However, a few students, mainly from nursing and allied health sciences, expressed dissatisfaction due to reduced practical classes and clinical exposure. Overall, the results indicate that online education methods played a major role in making learning purposeful and impactful during COVID-19.

Despite the successful continuation of education, a majority of students reported better understanding with traditional in-person teaching methods. This finding is consistent with previous studies showing higher satisfaction with traditional teaching due to enhanced engagement, motivation, and reduced distractions (34, 68). Various studies have compared the traditional method of anatomy teaching with online education during the COVID-19 pandemic. According to Cuschieri and Calleja Agius (15), Totlis et al. (69), and Sharma et al. (70), 56.00, 53.30, and 63.40% of students were satisfied with online education, respectively. Some studies also reported greater student satisfaction with traditional teaching methods compared to online lectures, citing disadvantages such as isolation and lack of teacher–student interaction (71, 72). However, variability in student satisfaction across studies indicates that contextual factors such as infrastructure, digital literacy, and socioeconomic conditions significantly influence the effectiveness of online education (69). The findings suggest that the majority of students favored theory classes delivered through the traditional teaching method over the online teaching method. The preference for in-person learning can be explained by the multisensory and immersive nature of anatomy education, which is difficult to replicate in a virtual environment.

A key component of the anatomy practical session involves the demonstration of models, specimens, histological slides, and hands-on cadaveric dissection. Hands-on cadaveric dissection is the most important part of anatomy practical teaching (55, 73). Franchi (74) described the impacts of the pandemic on anatomy practical education and highlighted how the lack of cadaveric dissection and hands-on anatomy practical experience may affect the future training of medical students. The reason for students’ preference for traditional methods in anatomy practical teaching might be the hands-on experience of dissection, which helps develop new skills and builds self-confidence.

Online anatomy education, although essential for maintaining academic continuity during the pandemic, revealed several inherent limitations that directly impact learning effectiveness. A major concern is the lack of student–teacher interaction, which contributes to reduced motivation, limited clarification of doubts, and a sense of isolation among students (69, 75). This highlights that learning in medical education is not solely content-driven but also depends on interactive and supportive learning environments, which are more effectively achieved through face-to-face engagement (29, 73, 76). Another critical limitation is the absence of cadaveric dissection and 3D learning experiences, which are fundamental for developing spatial understanding in anatomy (24, 55, 73, 74). The inability of virtual platforms to fully replicate these experiences underscores the continued importance of hands-on training in medical education. In addition, technical and infrastructural challenges, particularly ICI, significantly affect the accessibility and effectiveness of online learning, especially in developing regions (24, 66, 69, 77, 78). Limited digital literacy and awareness of online tools further compound these challenges (24, 79, 80). Environmental factors such as lack of a structured learning space, home distractions, and health concerns related to prolonged screen exposure also negatively influence student engagement and learning outcomes (5, 81–84). Taken together, these findings suggest that, while online teaching serves as a necessary alternative during crises, it is not sufficient as a standalone approach. The implications are clear: Future educational strategies must focus on improving digital infrastructure, enhancing interactive components, and integrating practical learning opportunities. This reinforces the need for hybrid or blended learning models that combine the flexibility of online education with the effectiveness of in-person teaching to ensure comprehensive and sustainable medical training.

Student satisfaction is a critical determinant of the effectiveness of online learning, as it directly influences engagement, comprehension, and overall learning outcomes (85, 86). Despite the rapid adoption of various online teaching methods during the pandemic, many students were unable to achieve all intended learning outcomes, particularly in anatomy, which relies heavily on practical exposure (73). This suggests that the limitation is not merely technological but also pedagogical, where the absence of interaction and hands-on experience reduces the depth of learning (87–89). Evidence from multiple studies indicates variable but generally low levels of student satisfaction with online education, reflecting its inconsistent effectiveness. Sharma et al. (70) reported a moderate satisfaction rate of 53.50%, whereas other studies showed significantly lower satisfaction levels, with only 14.00% of students satisfied in a broader review across medical disciplines (90–92). Similarly, Almendingen et al. (93) found that only 24.00% of students were satisfied, while the majority (76.00%) preferred returning to in-person education. These variations highlight that satisfaction is influenced by factors such as infrastructure, teaching methods, and discipline-specific requirements. The findings of the present study align with this broader trend, where dissatisfaction was primarily driven by reduced interaction and lack of hands-on training. Overall, these findings emphasize that, while online teaching was necessary during the pandemic, its long-term effectiveness depends on the integration of interactive elements and practical components, reinforcing the need for more adaptive and blended learning approaches in medical education.

Non-response rates for certain questions ranged from 1.30 to 17.90%. Although relatively small in percentage, these non-responses may indicate disengagement with certain survey topics or uncertainty among students, which could affect the accurate assessment of the true impact of negative experiences, particularly regarding the drawbacks of online learning.

### Future improvements in online anatomy theory classes

The need to improve online anatomy theory teaching primarily arises from the limited interaction and engagement inherent in virtual learning environments. Studies have emphasized that effective e-learning requires not only digital tools but also active student–teacher interaction to facilitate deeper understanding (33). This is reflected in the present study, where students emphasized the need for increased interaction, indicating that passive content delivery alone is insufficient. The demand for recorded lectures highlights the importance of flexibility in learning, allowing students to revisit complex topics and learn at their own pace. Similarly, the preference for small-group teaching indicates that students value more personalized and interactive learning environments, which can enhance participation and clarify doubts more effectively. The findings suggest that future strategies should move beyond simple content delivery and focus on interactive, student-centered teaching models, incorporating live discussions, small-group sessions, and recorded resources to optimize learning outcomes.

### Future improvements in online anatomy practical education

The need for improvement in online anatomy practical education is primarily driven by the inability of virtual platforms to replicate hands-on learning experiences (24, 74). The strong student response, advocating for live dissection and greater exposure to dissection halls, underscores the irreplaceable value of experiential learning in anatomy. The suggestion for recorded practical videos reflects the need for supplementary learning tools that can reinforce understanding and allow repeated visualization of complex anatomical structures (94). In addition, the preference for animation and 3D models highlights the role of advanced digital technologies in enhancing visualization, especially when direct access to cadaveric material is limited (95–97). Hence, the suggestions for improving online theory and practical anatomy education could be used to develop effective teaching strategies in the future. However, these findings collectively indicate that, while digital tools can enhance learning, they cannot fully substitute for hands-on experience. The key implication is that future anatomy education should adopt a hybrid approach, where traditional methods such as cadaveric dissection are complemented by digital innovations such as 3D visualization and recorded demonstrations. Such an approach would ensure both conceptual understanding and practical skill development, making anatomy education more resilient and adaptable in both routine and emergency scenarios.

Evidence suggests that a blended approach combining face-to-face and e-learning methods is the most effective model for future education (98). Globally, institutions have adopted advanced tools such as 3D anatomy software and digital learning platforms to enhance teaching (99–102). However, in low-resource settings, challenges such as limited device access, unstable electricity, and poor internet connectivity continue to hinder effective implementation (69). In the present study, students emphasized the need for better internet connectivity and advanced digital tools, while a small proportion of students highlighted the importance of awareness regarding online technologies and improved online assessment methods for theory and practical components in the future (103, 104). Overall, these findings highlight that, while digital tools are essential, their effectiveness depends on accessibility, infrastructure, and integration with traditional methods. This underscores the need for hybrid teaching models incorporating 3D visualization and in-person learning, which can support more flexible, resilient, and effective anatomy education in the future.

### Limitations of the study

This study has certain limitations. It was conducted at a single institution, which may limit the generalizability of the findings to settings with different technological resources or teaching practices. The study also did not stratify students by rural–urban background, which may have masked disparities in access to online learning, nor did it separately analyze undergraduate and postgraduate responses, potentially overlooking group-specific differences. Future studies incorporating demographic comparisons, objective assessments, and multi-institutional sampling are needed to provide more comprehensive insights into improving online and blended anatomy education. The study was not fully anonymous, as limited identifiers (name and sex) were collected.

## Conclusion

Anatomy is a fundamental pillar of medical education, essential for the development of clinical and surgical skills. The COVID-19 pandemic disrupted traditional teaching, leading to widespread adoption of online learning. This study highlights that, although online methods ensured academic continuity, they cannot fully replace conventional anatomy teaching. In the post-COVID era, student preferences for blended and hybrid learning models have directly influenced contemporary pedagogical practices, reinforcing the integration of flexible, technology-supported instruction as a standard component of present-day education.

Students emphasized the importance of teacher–student interaction and the continued relevance of hands-on cadaveric dissection, supported by increased exposure to dissection halls. Digital tools such as 3D models, animation, and recorded lectures and practical videos were valued for enhancing self-directed learning and conceptual clarity. The findings support the adoption of hybrid teaching models integrating traditional methods with digital innovations. Effective implementation requires robust digital infrastructure, reliable internet access, updated software, and faculty training in online pedagogy. In addition, addressing disparities in resource access is crucial, particularly in diverse socioeconomic settings. Collectively, these insights provide a framework for developing resilient, inclusive, and high-quality anatomy education systems adaptable to both routine and crisis situations.

## Data Availability

The original contributions presented in the study are included in the article/Supplementary material, further inquiries can be directed to the corresponding author/s.

## Glossary

MBBS: Bachelor of Medicine and Bachelor of Surgery
BDS: Bachelor of Dental Surgery
BOT-BPT: Bachelor of Occupational Therapy and Bachelor of Physiotherapy
PPT: PowerPoint presentation
YV: YouTube videos
SA: Software of Anatomy
eB: eBooks
SMP: Social media platform
All: All of the above
NR: No response
YUV: YouTube dissection videos
DA: Dissection atlas
3DDV: 3D dissection videos
HA: Histology atlas
3DM: 3D models
All: All of the above
NP: No practical
LS: In-person live lectures with live streaming
NoLS: In-person live lectures without live streaming
PRL: Previously recorded lectures
OLF: Other lecture format
WC: Whole class
SG: Small group
LWPS: Lectures as a whole group and practicals in small groups
LWNP: Lectures as a whole group and no practicals
HF: Helpful
NHF: Not helpful
PICT: Poor interaction and communication with teachers
LLD3D: Lack of live dissection and 3D models
ICI: Internet and connectivity issue
LAOT: Lack of awareness about online platform
LPTA: Lack of a proper teaching ambience
HRI: Health-related issues
ND: No drawback
NOTT: No online time-to-time test
IM: Improved
D: Deteriorated
NC: No change
BID: Both improved and deteriorated
MITPT: More interaction with teachers with a proper timetable of classes
NTINAS: Network and technical issues need to be resolved; new applications and anatomy software are required
RLRF: Recorded lectures for revision in the future
OOC: Only in-person classes
TLSGD: Theory lectures in small group discussions
OEA: Online exams or assignments
AONP: Awareness of online networks and platforms
LDED: Live dissection and more exposure to dissection
3DMA: 3D models and animation
RPV: Recorded practical videos
OOP: Only in-person practicals
SGP: Small-group practicals
MOE: More online exams
NIAS: Network issues need to be resolved, and new anatomy software should be used
AIOP: Awareness about the internet and online platforms
