# Supplementary material for: Impact of COVID-19 on anatomy education: a student-based survey and future perspectives
Source: Front Med (Lausanne). 2026 May 28;13:1700678. doi: 10.3389/fmed.2026.1700678 (PMC13254710; doi:10.3389/fmed.2026.1700678)
Supplement: Supplementary file 1 [file Data_Sheet_1.docx]

**Supplementary File**

**Impact of COVID-19 on Anatomy Education: A Student-Based Survey and Future Perspectives**

**Kapil Kumar Malviya^a*^**

^a^Department of Anatomy, Institute of Medical Science, Banaras Hindu University, Varanasi, U.P., India

* Corresponding authors:

Dr. Kapil Kumar Malviya

Associate Professor

Department of Anatomy

Institute of Medical Sciences

Banaras Hindu University

Tel.: +91 8052631840 (mobile).

E-mail addresses: [malviya2085@gmail.com](about:blank), [kkmalviya@bhu.ac.in](about:blank)

There were fifteen different questions related to anatomy education during the COVID-19 pandemic given as below along with multiple choice answers wherever applicable:

1) Name

2) Age/sex

3) Institution

4) Were you taught anatomy as a subject during COVID-19 pandemic? 1. Yes 2. No

if they selected yes, then they were eligible to solve the next eleven questions.

5) What was the mode of anatomy theory classes during the COVID-19 pandemic.

6) Please select from below the approaches used in online teaching classes for theory (you can select more than one option). (i) PowerPoint presentation (PPT) (ii) YouTube videos (iii) Software of anatomy (iv) eBooks (v) social media platform (vi) All the above.

7) Please select from below the approaches used in online teaching classes for practical (you can select more than one option). (i) You tube dissection videos (ii) Dissector Atlas (iii) 3D dissection videos (iv) Histology Atlas (v) 3D printing (vi) All the above.

8) For anatomy teaching through online lectures, which model in your opinion was best suitable (i) In person live lecture with live streaming (ii) In person live lecture without live streaming (iii) Previously recorded lectures (vi) Other lecture formats.

9) Were the online lectures and practical performed in small groups or whole class together; 10). Were the lectures taken in COVID-19 by using above mentioned tools (questions 6,7,8) helpful or not?

11) Were the online platforms for theory used during the pandemic were better and more understandable than the previous classical methods used in the pre-pandemic times?

12). Were the online platforms for practical used during the pandemic were better and more understandable than the previous classical methods used in the pre-pandemic times?

13) Please state some drawbacks of online teaching methods used during the pandemic.

14) Has medical education improved or deteriorated during COVID-19 pandemic?

15) In future what other methods can be used to improve online teaching and make it more successful if the pandemic persists or continues in future?

**Code Description**

**Q13.** Please state some drawbacks of online teaching methods used during the pandemic.

Various responses were obtained and were coded for explaining the drawbacks of online teaching methods as given: less interaction and communication with the teacher resulting in poor clearing of doubts and understanding (A), lack of live dissection and 3D models (B), internet issues and connectivity issues (C), lack of awareness about online platforms (D), lack of proper teaching ambience resulting in poor motivation, less interest, and less concentration (E), health related issues (F), no online time to time test (G) and no drawback (H).

**Q15.** In future what other methods can be used to improve online teaching and make it more successful if the pandemic persists or continues in future?

The responses obtained were divided into theory and practical-based responses

Participants could write more than one opinion in response to the question.

**1.** The theory-based response obtained from students were divided into seven codes: more interaction with teachers with the proper timetable of classes (A), network issues (B), the technical issues that need to be resolved and providing new applications and new anatomy software (C), provided recorded lectures for revision in future (D), only in-person classes needed (E), theory lectures in small group discussion (F), online time to time examination or provide assignments (G), and awareness about online network and platform needed (H).

**2.** Students gave various suggestions for the improvement of virtual practical teaching. The suggestions were summarized as: live dissection and more exposure to dissection hall (A), use of 3D models and animation for practices (B), providing recorded practical videos (C), only offline practices (D), small group practices (E), more online exams (F), network issue need to be resolved and use new anatomy software (G), provide awareness about internet and online platform (H).

**Exemplar coatings for each major code**

**Q13.**

less interaction and communication with the teacher resulting in poor clearing of doubts and understanding (A): Lack of realistic one-to-one interaction between teacher and students, Student teacher communication was minimal

lack of live dissection and 3D models (B): Practical not understandable without live demonstration, No dissection

Internet issues and connectivity issues (C): Classes were interrupted many times due to network problem, Light issue in rural area

lack of awareness about online platforms (D): In rural area lack of awareness about online platform, Lack of awareness regarding online platform

lack of proper teaching ambience resulting in poor motivation, less interest, and less concentration (E): Less concentration due to homely ambience, Loss of concentration and interest in the lecture as it gets monotonous, laziness in home environment

health-related issues (F): Headache due to continuous screen time, Eye straining

no online time-to-time test (G): Lack of time-to-time assessment of students

**Q15.**

**Theory-based response**

More interaction with teachers with the proper timetable of classes (A): Use of whiteboard in online classes instead of ppt, Proper schedule should be given for every class

Network issues (B): Proper network, Good network

The technical issues that need to be resolved and providing new applications and new anatomy software (C): Use better new applications for teaching, Technical issues should be cleared before the class.

Provided recorded lectures for revision in future (D): prerecorded videos, Videos should be recorded and sent to the students after class for revision.

Only in-person classes needed (E): only offline class, Classical offline,

Theory lectures in small group discussion (F): Dividing class into batches so that students can be monitored, Small group discussion

Online time-to-time examination or provide assignments (G): Online test, Regular test and assignment,

Awareness about online network and platform needed (H): Good network awareness, Awareness regarding use of online platform

**Q15.**

**Practical-based response**

live dissection and more exposure to dissection hall (A): Live dissection

Use of 3D models and animation for practices (B): Use of 3D anatomy models for better understanding, 3D models and Atlas,

Providing recorded practical videos (C): YouTube dissection videos, Recorded practical videos,

Only offline practicals (D): No online practicals, only offline practicals,

Small group practicals (E): Whole batch should be divided in small groups to make better demonstration.

More online exams (F): Examinations after topic completion, Regular test and assignment

Network issue need to be resolved and use new anatomy software (G): Anatomy software containing 3D images with an explanation of different anatomical structures, Better networking infrastructure

Provide awareness about internet and online platform (H): Awareness regarding use of online platform
